# A weighted energy consumption minimization-based multi-hop uneven clustering routing protocol for cognitive radio sensor networks

**DOI:** 10.1038/s41598-022-18310-9

**Published:** 2022-08-18

**Authors:** Jihong Wang, Conghui Li

**Affiliations:** School of Electrical Engineering, Northeast Electric Power University, Jilin, 132012 China

**Keywords:** Information technology, Electrical and electronic engineering

## Abstract

Aiming at solving the effective data delivery and energy hole problem in multi-hop cognitive radio sensor networks (CRSNs), a weighted energy consumption minimization-based uneven clustering (ECMUC) routing protocol is proposed in this paper. For the first time, the impact of control overhead on the network performance is taken into consideration, to be specific, the energy consumption of control overhead is integrated with that of data communication to model the network energy consumption. Through effective transformation and theoretical analysis, cluster radius of each ring is derived by minimizing the network energy consumption and balancing the residual energy among nodes in different rings. Distributed cluster heads (CHs) selection and cluster formation are carried out within this range to control the cluster size and the corresponding energy cost. Expected times for being CHs metric is defined to measure nodes’ energy and spectral potential and help select powerful CHs. Simulation results show that ECMUC protocol is superior to most clustering protocols designed for CRSNs in terms of network surveillance capability and network lifetime, and it is also demonstrated that taking control overhead into consideration is beneficial for improving the network performance.

## Introduction

Cluster-based cognitive radio sensor networks (CRSNs) are promising solutions to solve the energy efficiency and spectral efficiency problem faced by legacy wireless sensor networks (WSNs)^1^. Cognitive radio technology allows sensors to exploit licensed spectrum in an opportunistic manner and relieves the performance degradation incurred by spectrum shortage in unlicensed bands^2^. Due to the introduction of CR, the definition of neighbors has changed, i.e., two nodes are neighbors if they can satisfy the Euclidean distance requirement and share at least one common available channel. However, CR functions such as spectrum sensing and dynamic spectrum access will consume limited node energy, and the energy constraint problem of CRSNs is further aggravated. Therefore, minimizing the network energy consumption becomes the primary goal of protocol design for CRSNs. Clustering logically groups similar nodes in proximity and reduces traffic through data fusion and aggregation, which can help conserve energy^3^. In large-scale CRSNs, all nodes cannot reach the sink through single-hop communication, and multi-hop inter-cluster routing problem needs to be solved. Therefore, multi-hop clustering routing protocol design for CRSNs has become a hot topic in academia and industry.

The limitations of current research on multi-hop clustering routing protocol design for CRSNs can be summarized as follows:

- Current literature just focuses on the energy consumed by data communication and ignores the control overhead incurred during cluster heads (CHs) selection, cluster formation and route selection when theoretically analyzing the optimal number of clusters. Control information exchange will bring non-negligible energy consumption to the whole network^4^. Ignoring this part of energy consumption is beneficial for simplifying the theoretical analysis, but the obtained results may be inappropriate for achieving the design goal.
- Most existing clustering protocols for CRSNs are uniform clustering protocols which establish clusters with almost the same size. Due to the towards-the-sink type of traffic in CRSNs, CHs close to the sink need to perform more inter-cluster relay forwarding tasks which will accelerate their energy exhaustion. The energy hole problem incurred by uneven distribution of energy consumption among CHs will occur^5^. In addition, the early death of CHs which are close to the sink and network partition will significantly degrade the surveillance capability of CRSNs^6^.
- Current uneven clustering protocols proposed for CRSNs usually adopt constant coefficients, and the cluster radius is designed to linearly increase as the Euclidean distance to the sink increases. However, the optimality of the simple linear relationship has not been proven, and the coefficients should be analyzed and optimized according to specific network configurations instead of fixing their values. Here, the specific network configurations refer to the network size, node density, maximum node transmission range, data packet size, control packet size and Euclidean distance to the sink.

In order to take full advantages of uneven clustering, i.e., enabling the CHs close to the sink to conserve more energy for inter-cluster relay and solving the energy hole problem effectively^7^, a weighted energy consumption minimization-based uneven clustering (ECMUC) routing protocol is proposed in this paper. By minimizing the network energy consumption and balancing the residual energy among nodes in different rings, the optimal cluster radius under specific network configurations is derived. ECMUC partitions the whole CRSNs into uniform rings and forms uneven clusters based on the obtained cluster radius within these rings. The innovations of ECMUC protocol are summarized as follows:

- Energy consumption of data communication and control overhead are for the first time integrated to model the network energy consumption, and minimizing it is the primary design goal of ECMUC protocol.
- The objective of minimizing the network energy consumption and balancing the residual energy among nodes in different rings to solve the energy hole problem is innovatively transformed into minimizing the weighted sum of the energy consumption of each ring and the additional energy consumption introduced to the whole network by it. Through theoretical analysis, cluster radius in each ring is reasonably set to control cluster size.
- Expected times for being CHs (ETBCHs) metric is defined and leveraged to measure nodes’ energy and spectral potential. Based on ETBCHs comparison within the cluster radius, the most powerful node in the neighborhood becomes a final CH. ECMUC protocol can autonomously achieve distributed CHs selection, cluster construction and multihop inter-cluster route selection to forward the monitored data towards the sink. Extensive simulations show that ECMUC protocol can significantly improve the network surveillance capability and extend the network lifespan.

The rest of this paper is organized as follows. “Related work” section reviews clustering protocols designed for CRSNs, including uniform and uneven clustering protocols. System model is described and ECMUC protocol is explained in details in “Methods” section. “Results and discussion” section tests and evaluates the performance of ECMUC protocol. Finally, “Conclusions” section concludes the paper and points out our future research directions.

## Related work

According to the data report model adopted, current clustering protocols for CRSNs can roughly be classified into event-driven clustering protocols and time-triggered clustering protocols. Event-driven clustering protocols, such as ESAC^8^, mESAC^9^ and ERP^10^, are triggered by emergent events and form temporary clusters in the corridor between the event and the sink until the event ends up. They are designed for event-driven applications and are not suitable for applications which require continuous and periodical information collection^11^. On the contrary, time-triggered clustering protocols form clusters in the whole network and maintain them by periodical calculation and communication until the end of network lifetime. They can be further divided into uniform clustering protocols and uneven clustering protocols based on the cluster size. In the following sub-sections, we will review related work from these two aspects.

### Uniform clustering protocols for CRSNs

Uniform clustering protocols for time-triggered CRSNs can be further divided into 3 sub-categories, i.e., centralized, distributed and hybrid protocols. CogLEACH-C^12^, ABCC^13^ and IMOCRP^14^ are typical representatives of centralized protocols. Though different factors are considered when selecting CHs, necessary information should be collected and handled by the sink, which requires that all CRSNs nodes should reach the sink through single-hop communication. Therefore, they all suffer problems such as network scalability and single point of failure, which limits their application field.

Most clustering protocols for time-triggered CRSNs are distributed and uniform cluster-based in nature, such as CogLEACH^15^, NSAC^16^, EACRP^17^, ESUCR^18^ and so on, as shown in Table 1. These protocols select CHs and form clusters in the locality through extensive information exchange. Among them, CogLEACH is a distributed spectrum-aware extension of LEACH protocol^19^. CogLEACH uses the number of idle available channels as probability weight for CHs selection, and each CRSNs node can independently judge whether itself can become a CH or not by comparing its CHs weight with a random number. NSAC builds stable clusters by taking the spectrum dynamics and energy consumption comprehensively into consideration. Nodes with the highest weight in the neighborhood win competition and become CHs, and other nodes in the maximum edge biclique become corresponding cluster members (CMs). These nodes will be excluded from clustering, and cluster formation continues until all CRSNs nodes are clustered. EACRP and ESUCR iteratively merge neighboring clusters until the optimal number of clusters is achieved. Except for the residual energy, the number of available channels, the number of neighbors and the distance to the sink are used to compute CHs weight. Primary and secondary gateway nodes are leveraged to relay packets between neighboring CHs.

**Table 1:** Characteristics analysis of existing clustering protocols for CRSNs.

Protocols	Type	Scenarios	Control overhead		Inter-cluster routing	Objective
			Clustering	Routing		
CogLEACH-C	C	SHop-All	3* N*-*K*	0	×	×
ABCC	C	SHop-All	*N*	0	×	MinASDE
IMOCRP	C	SHop-All	*N*	0	×	MinASDE
CogLEACH	D	SHop-CHs	2* N*	0	×	×
NSAC	D	SHop-CHs	–	0	×	×
EACRP	D	MHop	3* N* × *ite*_*EACRP*_	–	√	×
ESUCR	D	MHop	*4 N* × *ite*_*ESUCR*_	–	√	×
WCM	H	SHop-CHs	4* N*	0	×	×
LEAUCH	D	MHop	2* N* + 2*N*_*candi*_	–	×	×
R-bUCRP	D	SHop-CHs	*N* + *2N*_*candi*_	0	×	×
IACUCAPTEEN	D	MHop	2* N* + *N*_*candi*_	–	√	×
ESAUC	D	MHop	*N* + *2N*_*candi*_	–	√	×

C: centralized, D: distributed, H: hybrid; SHop-All: single-hop communication between all nodes and the sink, SHop-CHs: single-hop communication between CHs and the sink, MHop: multi-hop communication; MinASDE: minimize the average node energy consumption and the standard deviation of node residual energy; *N* is the number of living nodes in current round; *K* is the optimal number of CHs; *ite*_*EACRP*_ and *ite*_*ESUCR*_ are the number of merging iterations performed by EACRP and ESUCR, respectively; *N*_*candi*_ is the number of candidate CHs in uneven clustering protocols; ― represents that the corresponding value is unable to be explicitly quantified; × denotes the corresponding problem has not been solved while √ represents the opposite situation.

WCM-based spectrum-aware clustering protocol (hereinafter refer to as WCM)^20^ is a typical case of hybrid clustering protocols. In WCM, a new weighted metric which simultaneously evaluates the temporal-spatial correlation, confidence level and residual energy is proposed to select CHs. Then the sink merges neighboring clusters with the highest temporal-spatial correlation until the objective function cannot be improved any more. Extensive control information exchange will quickly drain node battery, and then nodes will die.

In all uniform clustering protocols, besides intra-cluster data communication, CHs near the sink should also relay packets for other parts of the network, which means balanced energy distribution among CHs cannot be guaranteed. Therefore, early death of CHs near the sink will occur and this will further result in network partition.

### Uneven clustering protocols for CRSNs

Different from above uniform clustering protocols, cluster radius in uneven clustering protocols increases as the distance to the sink increases, which can help balance the residual energy among CHs. LEAUCH^21^ is a representative uneven clustering protocol for CRSNs, and it determines final CHs by competition among candidate CHs within the cluster radius. Cluster radius is calculated according to the Euclidean distance to the sink *d*_*i*,*sink*_, as shown in Eq. (1).

1 $$R_{ci} = \left( {1 - c\frac{{d_{max} - d_{i,sink} }}{{d_{max} - d_{min} }}} \right)R_{c}^{0}$$

where *R*_*c*_^0^ is the maximum cluster radius of candidate CHs; *c* is a constant coefficient for uneven clustering; *d*_*max*_ and *d*_*min*_ are the maximum and minimum Euclidean distance to the sink from CRSNs nodes, respectively. However, the candidate CHs selection threshold 0.4 has a large impact on network performance, and in most cases, no candidate CHs can be selected at all. R-bUCRP^22^ adopts the same manner to calculate cluster radius, and its random candidate CHs selection cannot guarantee the optimal distribution of candidate CHs. Therefore, the CHs selection is largely affected. IACUCAPTEEN^23^ and ESAUC^24^ improve the cluster radius calculation of LEAUCH by taking more factors into consideration, such as the node residual energy, the number of neighbors and the number of available channels. However, they still adopt fixed weighted coefficients which need to be analyzed and optimized according to specific network configurations. In addition, the weighted coefficient *ω* in ESAUC is not given.

As stated above, current uneven clustering protocols are basically proposed on the basis of LEAUCH, therefore, cluster radius is not obtained by theoretical derivation with the purpose of balancing the residual energy among CHs. Instead, the cluster radius is designed to linearly increase as the Euclidean distance to the sink increases, which needs to be analyzed and optimized^25^.

From above table and analysis, we can obtain the following observations:

- Current research on theoretical analysis of the optimal number of clusters wholly focuses on the energy consumption of data communication, while the energy cost of control information exchange is neglected. In addition, in order to simplify analysis, current literature usually simplifies the calculation process of energy consumption of inter-cluster communication, that is to say, assumptions are made that each data packet needs at most once relay to reach the sink. Actually, multi-hop inter-cluster relay is more practical, especially in large-scale CRSNs. This may restrict the network scalability and lead to the inapplicability of the derived results.
- Current uniform clustering protocols cannot guarantee balanced energy distribution among CHs. Therefore, early death of the CHs which are close to the sink will occur and this will further result in network partition.
- In existing uneven clustering protocols for CRSNs, the cluster radius is usually quantified under given constant coefficients, and it is assumed to be linearly proportional to the Euclidean distance to the sink. However, in order to take full advantages of the potentials of uneven clustering in balancing the residual energy among CRSNs nodes, these coefficients and cluster radius should be optimized according to specific network configurations.

All these motivate us to propose ECMUC protocol which integrates energy consumption of data communication and control information exchange together for the first time and solves the energy hole problem effectively by balancing the residual energy among nodes in different rings as much as possible.

## Methods

### System model

*N* homogeneous CRSNs nodes with initial energy *E*_0_ are uniformly and randomly distributed in the surveillance area (the area is approximated as a circle), coexisting with *P* randomly-deployed primary users (PUs). The sink is located at the center to gather useful information about the whole area and provides it to end users^26^. Once deployed, CRSNs nodes no longer move unless their residual energy is exhausted. Each CRSNs node is configured with one transceiver, so it cannot send out and receive information at the same time. Additionally, it leverages received signal strength or certain localization algorithm to obtain its relative distance from the receiver, according to which it can adapt its transmission power. Competition-based method is applied for CHs selection, and the most qualified node in the neighborhood becomes final CH who is responsible for intra-cluster data collection and aggregation. The data aggregation effect of CHs can be measured by aggregation coefficient *β*. Here, perfect aggregation is assumed, which means packets from CMs can be fused into a single packet with fixed length, i.e., *β* is equal to the reciprocal of the number of CRSNs nodes in the whole cluster^27^. Restricted by limited node transmission range, multi-hop communication is exploited to realize inter-cluster data delivery.

**Figure 1:**
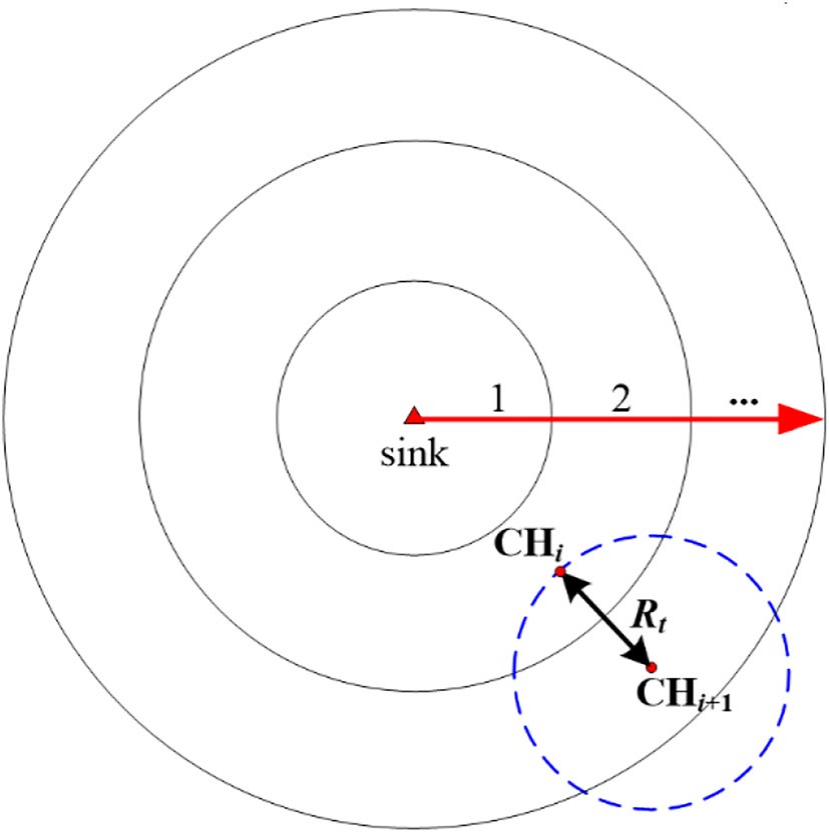
Uniform ring division with ring width *R*_*t*_.

In order to determine cluster membership based on the Euclidean distance to the sink, the surveillance area is partitioned into different rings around the sink. Uneven ring division usually results in more rings, especially for large-scale CRSNs. More rings mean more times of inter-cluster data relay which will lead to heavier energy consumption. Therefore, in order to reduce energy consumption and guarantee the minimum data transmission delay, we prefer to organize our CRSNs into uniform rings whose width is *R*_*t*_, as shown in Fig. 1.

Each CRSNs node consumes energy in sending and receiving control and data packets, and the energy consumption of transmitting an *l*-bit packet over distance *d*_*j*,*k*_ is quantified by^19^:

2 $$E_{TX} = \left\{ \begin{gathered} l \times (E_{elec} + \varepsilon_{fs} d_{j,k}^{2} )\quad if\;d_{j,k} \le d_{0} \hfill \\ l \times (E_{elec} + \varepsilon_{mp} d_{j,k}^{4} )\quad otherwise \hfill \\ \end{gathered} \right.$$

where *E*_*elec*_ is the energy consumption of transceiver electronics per bit; *ε*_*fs*_ and *ε*_*mp*_ are the energy consumption of power amplifier per bit in free-space and multi-path loss model, respectively; *d*_0_ is the distance threshold, and *d*_0_ = (*ε*_*fs*_/*ε*_*mp*_)^1/2^. The energy consumption of receiving corresponding packet is:

3 $$E_{RX} = l \times E_{elec}$$

Markov renewal process is widely used in literature to imitate PUs activity, and it assumes ON/OFF model for PUs behavior^28^. In other words, PUs activity on given channel alternates between ON and OFF states whose duration are geometrically distributed random variables with parameters *p*_*c*_ and *q*_*c*_.

### Weighted energy consumption minimization-based multi-hop uneven clustering routing protocol design for CRSNs

As mentioned earlier, our design goal is to minimize the energy consumption of all rings in CRSNs and balance the residual energy among nodes in different rings to avoid energy holes. The energy hole problem occurs due to the uneven distribution of energy consumption among nodes in different rings, i.e., nodes in inner rings drain their battery more quickly than those in outer rings. Therefore, in order to solve this problem, ECMUC must slow down the node death rate of inner rings to guarantee that it should not be faster than outer rings. Based on uniform ring division with fixed ring width *R*_*t*_, our clustering objective can be transformed into minimizing the weighted sum of the energy consumption of ring *i* (*i* ∈ [1, 2, …, *L*_*max*_]) and the additional energy consumption brought to the whole network by it. Here, *L*_*max*_ is the maximum ring number of all CRSNs nodes, as shown in Eq. (4).

4 $$L_{max} = \left\lceil {\frac{{\mathop {\max }\limits_{\forall \,j \in [1,2, \cdots ,N]} \;\sqrt {(x_{j} - x_{sink} )^{2} + (y_{j} - y_{sink} )^{2} } }}{{R_{t} }}} \right\rceil$$

where *N* is the total number of CRSNs nodes; (*x*_*j*_, *y*_*j*_) and (*x*_*sink*_, *y*_*sink*_) are the coordinates of node *j* and the sink, respectively. *R*_*t*_ is the maximum transmission range of CRSNs nodes. Each node *j* determines its ring index according to Eq. (5) below.

5 $$L(j) = \left\lceil {\frac{{d_{j,sink} }}{{R_{t} }}} \right\rceil$$

where *d*_*j*,*sink*_ is the Euclidean distance from node *j* to the sink. *L*(*j*) will be used for CHs selection and cluster formation later on.

In order to achieve our clustering objective, ECMUC enables all CRSNs nodes in ring 1 to act as separate CHs and share the inter-cluster data forwarding tasks, which can help reduce their energy consumption speed. In addition, ECMUC balances the residual energy among nodes in different rings by reasonably setting cluster radiuses, and we will show how to determine their values through theoretical analysis according to specific network configurations. Of course, ECMUC also tries to balance the residual energy of nodes in the same ring as much as possible, such as CHs rotation among nodes in ring *i* (*i* ≠ 1) based on the ETBCHs metric or enabling nodes in ring *i* (*i* ≠ *L*_*max*_) to take turns acting as relays. ETBCHs metric is defined by combining the energy potential and channel availability of CRSNs nodes. Based on this metric, CHs competition in the neighborhood is carried out to determine final CHs who are responsible for building their own clusters and search for available inter-cluster routing paths.

### Design details of ECMUC protocol

In CRSNs, due to the dynamic channel occupancy behavior of PUs, the channel availability is time- and position-dependent. At the beginning of each round, each CRSNs node *j* leverages CR functions such as spectrum sensing to perceive the channel availability at its position and decides its available channel list **C**_***j***_, which determines whether CRSNs nodes can communicate with each other and whether information transmission can be successful. CHs selection, cluster formation and route selection of ECMUC are all based on the available channel information. In other words, channel availability obtained from CR functions will affect the clustering results and the next-hop relay selection, which will further affect node energy consumption. In ECMUC, CHs competition is conducted among neighboring nodes belonging to the same ring, and different strategies will be applied for distinct *L*_*max*_. To be specific, if all CRSNs nodes can reach the sink through single-hop communication, i.e., *L*_*max*_ = 1, energy consumption of the whole network can be minimized by setting each CRSNs node as a separate CH. In other words, non-clustering is the most energy-efficient way of network organization in this scenario^14^. For example, if data from *n* normal nodes needs to be transmitted to the sink, for simplicity reason, distance from each node to the sink *d*_*tosink*_ is assumed to be the same and *d*_*tosink*_ ≤ *d*_0_.

**Figure 2:**
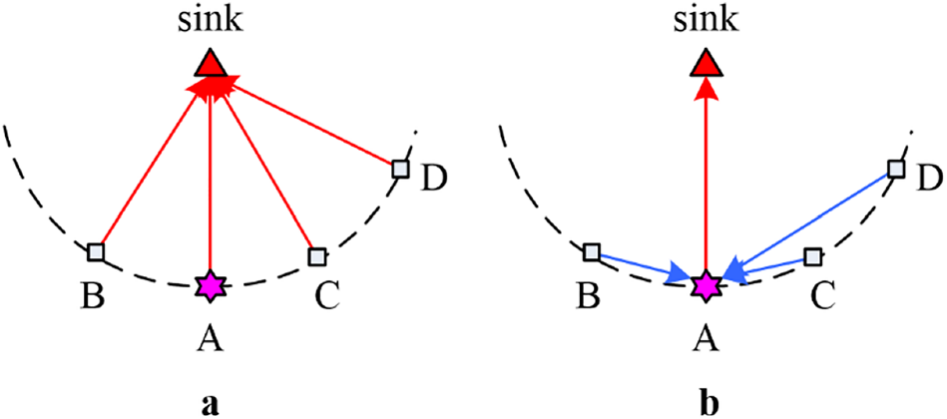
Comparison of energy consumption in non-clustering and clustering scenarios.

Without clustering (as shown in Fig. 2a), the total energy consumption *Etotal* is:

6 $$E_{total} = n \times (E_{elec} + \varepsilon_{fs} d_{tosink}^{2} ) \times l$$

In clustering case, assuming that data from other nodes is aggregated at node A and then the aggregated data is transmitted to the sink, as shown in Fig. 2b. The total energy consumption *E*_*total*_*'* is:

7 $$E_{total}^{\prime } = \sum\limits_{i = 1}^{n - 1} {(E_{elec} + \varepsilon_{fs} d_{{i,{\text{A}}}}^{2} ) \times l} + (n - 1) \times E_{elec} \times l + n \times E_{DA} \times l + (E_{elec} + \varepsilon_{fs} d_{tosink}^{2} ) \times l$$

where the first item on the right side is the total energy consumption of data transmission from other nodes to A. The second item is the energy consumed by A in data reception. The third item is the energy cost of data aggregation at A, and the last item is the energy spent on data transfer from A to the sink.

The difference in the total energy consumption between above two cases is as follows:

8 $$\Delta E = E_{total}^{\prime } - E_{total} = l \times \left[ {(n - 1) \times E_{elec} + n \times E_{DA} + \sum\limits_{i = 1}^{n - 1} {\varepsilon_{fs} d_{i,A}^{2} - (n - 1) \times \varepsilon_{fs} d_{tosink}^{2} } } \right]$$

For typical transmission range of CRSNs nodes, it is usually the case that ∆*E* > 0, which means non-clustering can lead to lower energy consumption. This conclusion will be verified by the simulation results in Performance analysis in Case 1 section. In this case, each living CRSNs node does not need to consume extra energy to exchange control information for CHs selection and cluster formation. Instead, it sends its monitored data to the sink directly to minimize the total energy consumption. In particular, data transmission is performed on randomly selected channel from available channel list, and the purpose is to reduce the number of competing CHs who contend for channel access when carrier sense multiple access with collision avoidance (CSMA/CA) protocol is applied.

If all CRSNs nodes cannot reach the sink through single-hop communication, i.e., *L*_*max*_ ≥ 2, all CRSNs nodes in ring 1 still act as independent CHs. This enables more nodes to share the inter-cluster data forwarding tasks and reduce the energy consumption speed, which is beneficial for solving the energy hole problem. ETBCHs metric should be compared among neighbors in the same ring (except ring 1) to compete for CHs, and the one with the highest ETBCHs value in the neighborhood becomes final a CH. In round *t*, ETBCHs value of node *j* (in ring *i*) is defined as follows:

9 $$ETBCHs(j) = \left\lfloor {\frac{{E_{j} }}{{\frac{{E_{CH\_j(i)} + E_{sw} \times intra\_sw}}{{neigh_{j} + 1}}}}} \right\rfloor \times \log_{2} (1 + \frac{{\sum\limits_{k = 1}^{{c_{j} }} {n_{common\_k} } }}{{C \times neigh_{j} }})$$

where *E*_*j*_ is the residual energy of node *j*; *E*_*CH*_*j*(*i*)_ is the expected energy consumption of node *j* for being a CH once in ring *i*, and of course, it does not include the energy consumption of channel switching. Therefore, the energy spent on intra-cluster channel switching is further added. *E*_*sw*_ is the energy consumption per channel switching, and *intra_sw* is the expected minimum times of channel switching performed by *j*. *neigh*_*j*_ is the number of neighbors of node *j*. ⌊*x*⌋ returns the nearest integer which is smaller than or equal to *x*. As CHs role will rotate among node *j* and its neighbors as network operation goes on, *j* will become a CH every *neigh*_*j*_ + 1 rounds on average. Therefore, the first item in Eq. (9) quantifies the maximum number of times to be a CH from energy perspective for node *j*. If node *j* does not have enough energy for being a CH in this round, its ETBCHs value is zero. This can avoid unnecessary energy waste during cluster formation and data transmission. *c*_*j*_ is its number of available channels. *C* is the total number of licensed channels. *n*_*common*_*k*_ is the number of neighbors who share channel *k* with *j*. We all know that if node *j* has no available channel or it does not share common channels with its neighbors, i.e., $\sum\nolimits_{k = 1}^{{c_{j} }} {n_{common\_k} }$ = 0, it should not become a CH. In order to guarantee this, we add 1 to the log function. Actually, the log function item represents the expected probability of being a CH from channel connectivity perspective. Therefore, ETBCHs value measures the potential times of node *j* for being a CH by combining its energy potential and spectral potential.

**Figure 3:**
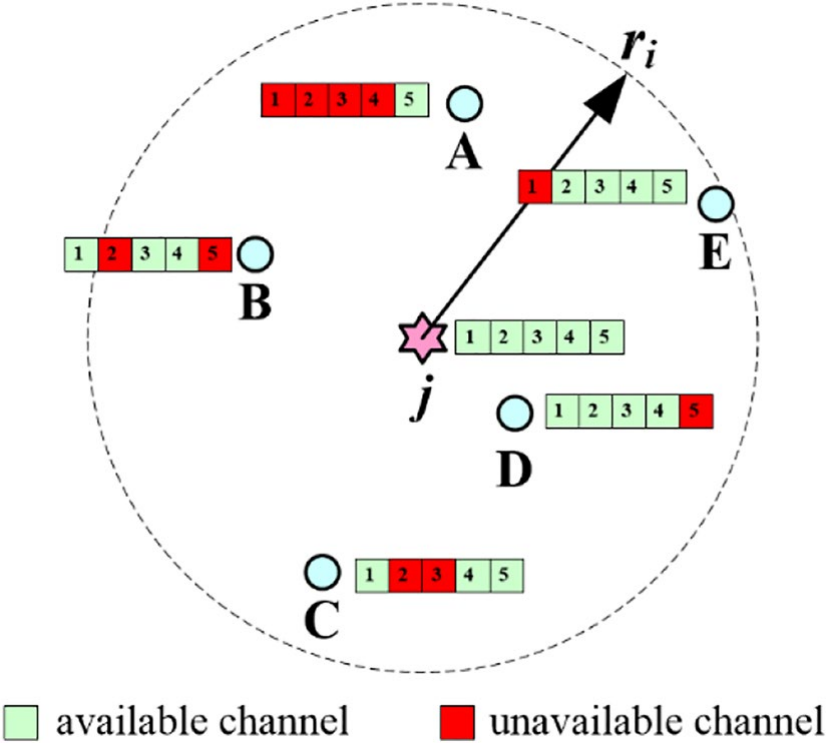
Example of acquiring parameters for ETBCHs calculation.

In order to calculate ETBCHs value, we need to obtain *c*_*j*_, *neigh*_*j*_, *E*_*CH*_*j*(*i*)_, *intra_sw* and *n*_*common_k*_. Among them, *E*_*CH*_*j*(*i*)_ can be obtained through analysis in Theoretical analysis of cluster radius *r*_*i*_ section, and we use Fig. 3 to illustrate how to acquire the values of other parameters.

Assuming that 5 nodes A, B, C, D and E are located within the cluster radius range (*r*_*i*_) of node *j*, we have *neigh*_*j*_ = 5. The bar with numbers next to each node shows the availability of all licensed channels. From Fig. 3, we can see that 5 licensed channels are all available to *j*, therefore, *c*_*j*_ = 5. Node *j* can reach nodes B, C and D on channel 1, therefore, *n*_*common_*1_ = 3. Similarly, *n*_*common_*2_ = 2, *n*_*common_*3_ = 3, *n*_*common_*4_ = 4, *n*_*common_*5_ = 3. If node *j* can become a final CH, it will broadcast time division multiple access (TDMA) schedule which assigns dedicated time slot and channel to each CM within *r*_*i*_. The CH will choose the channel which is available to majority of its CMs as cluster channel. If above channel is not available to a certain CM, the CH will select one common available channel between them to enable their data delivery. We call this hybrid constraint, which means that groupwise constraint^29^ is the main constraint and pairwise constraint^30^ is used as supplement. *intra_sw* is relevant to above intra-cluster schedule. In Fig. 3, as *j* can reach 4 neighbors on channel 4, channel 4 is specified as cluster channel. However, node A cannot use channel 4 in this round, therefore, CH *j* should select one common available channel to communicate with A, say channel 5. In order to minimize extra energy consumption brought by channel switching, the best schedule strategy for node *j* is scheduling transmissions from nodes B, C, D and E in 4 consequent time slots on channel 4, and then it switches to channel 5 to communicate with node A. Therefore, it performs once channel switching in this case, that is, *intra_sw* = 1.

**Figure 4:**
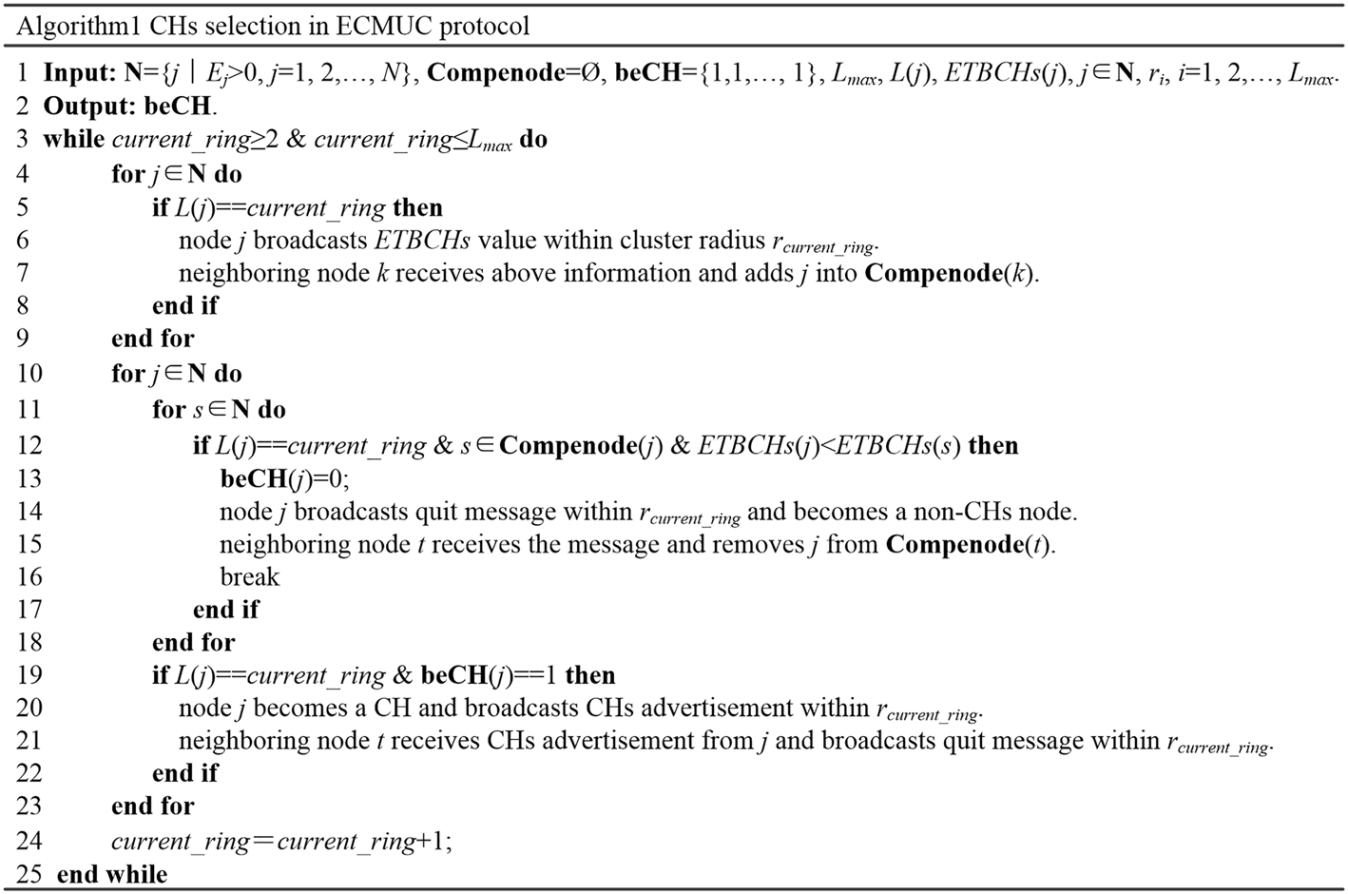
Pseudo code of CHs selection in ECMUC protocol.

After ETBCHs calculation, all living CRSNs nodes compete for CHs according to Algorithm1, as shown in Fig. 4.

Lines 4–9 show the exchange of ETBCHs value. Each living node *j* (*L*(*j*) ≠ 1) broadcasts its ETBCHs value within cluster radius *r*_*L*(*j*)_, and its neighbors who are located in the same ring add *j* into their competing node set.

Each node, say *j*, compares its ETBCHs value with its neighbors in its competing node set **Compenode**(*j*). If *ETBCHs*(*j*) is smaller than one of them, node *j* quits from CHs competition, becomes a non-CHs node and waits for joining one cluster as a normal CM. It broadcasts quit message within *r*_*L*(*j*)_ to notify its neighbors, and those who receive this message remove *j* from their competing node set, as shown in lines 11–18. Otherwise, if *ETBCHs*(*j*) is the highest in the neighborhood, *j* becomes a final CH and broadcasts CHs advertisement within *r*_*L*(*j*)_ to announce its role. The advertisement includes its node ID, residual energy and available channel list to provide sufficient information for normal nodes. Nodes in **Compenode**(*j*) quit from CHs competition on receiving the CHs advertisement, as shown in lines 19–22.

During CHs selection, control packets such as node information, ETBCHs value, quit message and CHs advertisement are broadcast within the cluster radius. The energy consumed by these packets largely depends on the value of the cluster radius, and we will analyze how to obtain its value in the Theoretical analysis of cluster radius *r*_*i*_ section.

After determining CHs in each ring, cluster formation simultaneously begins in all rings except ring 1. For each living non-CHs node, say *j*, it selects its CH according to the number of common available channels and the Euclidean distance between them. Node *j* sends out join request to the final CH and requests to become its member if such CH exists. The CH receives the join message and adds *j* into its CMs list. If such CH cannot be found, node *j* becomes a separate CH, and it will broadcast CHs advertisement similar to that in Algorithm1. Above process continues until all nodes are clustered.

For each CH, say *k*, if its ring index *L*(CH(*k*)) ≥ 2, it should select one CH (represented by CH(*s*)) from the next inner ring towards the sink to relay its packets. In order to provide information for next-hop relay selection, each CH whose ring index is larger than 1 but smaller than *L*_*max*_ broadcasts its own information such as residual energy and Euclidean distance to the sink within *R*_*t*_. The next-hop relay is chosen among candidate next-hop relays according to Eq. (10). Here, the candidate next-hop relay should satisfy the following conditions: *L*(CH(*s*)) = *L*(CH(*k*))-1 and *d*_CH(*k*),CH(*s*)_ ≤ *R*_*t*_.

10 $$W_{{{\text{CH}}(k)}} ({\text{CH}}(s)) = \left| {{\mathbf{C}}_{{{\text{CH}}(k)}} \cap {\mathbf{C}}_{{{\text{CH}}(s)}} } \right| \times E_{{{\text{CH}}(s)}} \times \frac{{E_{con} ({\text{CH}}{\kern 1pt} (k),{\text{CH}}{\kern 1pt} (s))}}{{E_{con} ({\text{CH}}{\kern 1pt} (s),sink)}}$$

where **C**_CH(*k*)_ and **C**_CH(*s*)_ are the available channel set of CH(*k*) and CH(*s*), respectively. The first item on the right side of Eq. (10) represents the number of common channels shared by CH(*k*) and CH(*s*). *E*_CH(*s*)_ is the residual energy of CH(*s*). *E*_*con*_(CH(*k*),CH(*s*)) is the energy consumption of data communication between CH(*k*) and CH(*s*), while *E*_*con*_(CH(*s*),*sink*) is the energy consumption of data transmission from CH(*s*) to the sink. Actually, the ratio of these two items helps select appropriate next-hop relay which is located near the sink and a little far away from CH(*k*). The inner CH with more common available channels, more residual energy and closer to the sink has higher probability of being selected. A notification message is sent to the next-hop relay to inform about the final routing decision. If such CH cannot be found, one of its CMs who is closer to the sink is chosen to help relay the data packet towards the next inner ring. It should be noted that all nodes in ring 1 transmit their information which includes the residual energy, available channels and so on through direct link to the sink. The purpose is to reduce the energy cost during route selection, as there are numerous CHs in ring 1. Then the sink will broadcast above information to CHs in ring 2, and these CHs can choose them as next-hop relays according to Eq. (10).

### Theoretical analysis of cluster radius

In this section, we will theoretically analyze how to set cluster radius *r*_*i*_ according to specific network configurations. Taking nodes in ring *i* (*i* ≠ 1) as an example, energy is consumed in the following 4 phases: CHs selection, cluster formation, route selection and data transmission.

**Figure 5:**
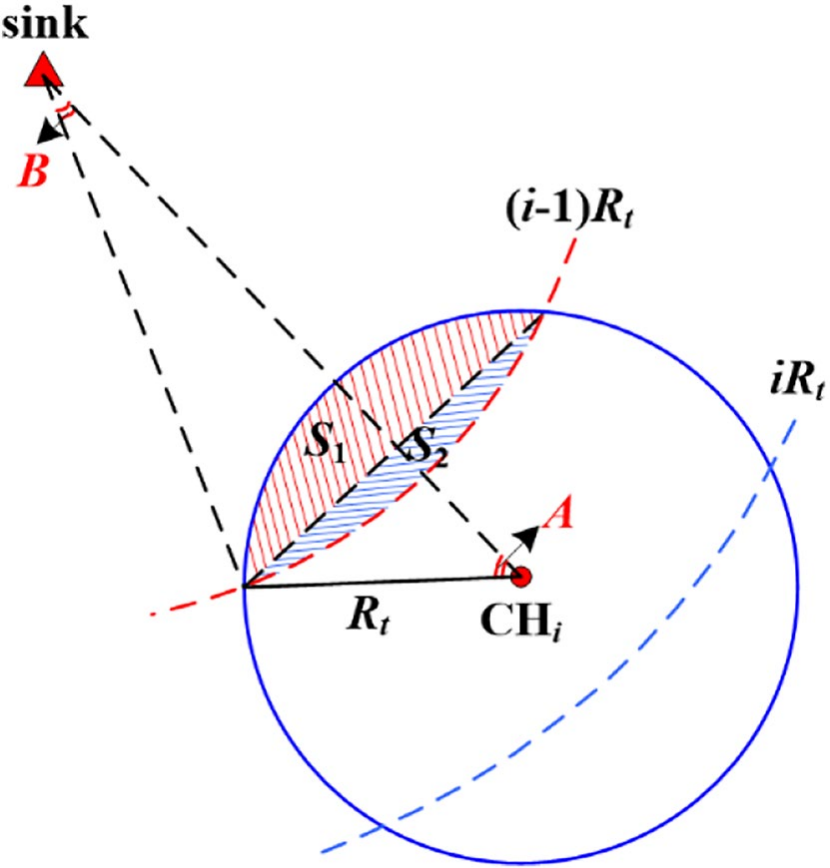
Illustration of *P*_*inRt*_ calculation.
  According to the Cosine Law, we have:
  17 $$A = {\text{arccos}}\frac{{R_{t}^{2} + \left[ {(i - \frac{1}{2})R_{t} } \right]^{2} - \left[ {(i - 1)R_{t} } \right]^{2} }}{{2 \times R_{t} \times (i - \frac{1}{2})R_{t} }} = {\text{arccos}}\frac{{i + \frac{1}{4}}}{{2(i - \frac{1}{2})}}$$
  Consequently, the arch area *S*_1_ is obtained by subtracting the area of the triangle from the area of corresponding sector, as shown in Eq. (18).
  18 $$S_{1} = \pi R_{t}^{2} \times \frac{A}{\pi } - \frac{1}{2}R_{t}^{2} \times \sin 2A = R_{t}^{2} \times (A - \frac{1}{2}\sin 2A)$$
  Similarly, we can obtain:
  19 $$S_{2} = \left( {i - 1} \right)^{2} R_{t}^{2} \times (B - \frac{1}{2}\sin 2B)$$
  Therefore, the probability *P*_*inRt*_ is:
  20 $$P_{inRt} = \frac{{S_{1} + S_{2} }}{{S_{Total\_i - 1} }} = \frac{{A - \frac{1}{2}\sin 2A + (i - 1)^{2} (B - \frac{1}{2}\sin 2B)}}{{\left( {2i - 3} \right)\pi }}$$
  The additional energy consumption brought to the whole network by ring *i* in route selection phase is:
  21 $$\Delta E_{Route} = K_{i} \times E_{elec} \times l_{1} + (E_{elec} + \varepsilon_{fs} R_{t}^{2} ) \times l_{1} \times K_{i - 1}$$
- The energy consumption of ring *i* and the additional energy consumption introduced to the whole network by ring *i* in data transmission phase.
  Each CH broadcasts TDMA schedule within its cluster, and it also receives and aggregates data from its members and then transfers the aggregated information to the sink. In addition, each CH should relay data packets from outer rings. Therefore, its energy consumption is calculated according to Eq. (22):
  22 $$e_{Data\_CH} = (E_{elec} + \varepsilon_{fs} r_{i}^{2} ) \times l_{1} + (n_{i} - 1) \times E_{elec} \times l + n_{i} \times E_{DA} \times l + (E_{elec} + \varepsilon_{fs} R_{t}^{2} ) \times l + (2E_{elec} + \varepsilon_{fs} R_{t}^{2} ) \times l \times \frac{1}{{K_{i} }}\sum\limits_{j = i + 1}^{{L{}_{max}}} {K_{j} }$$
  Each CM receives the TDMA schedule from its CH and transmits data according to the schedule. Its energy cost in this phase is:
  23 $$e_{Data\_CM} = E_{elec} \times l_{1} + (E_{elec} + \varepsilon_{fs} d_{toCH}^{2} ) \times l$$
  We can obtain the energy consumption of ring *i* in data transmission phase as follows:
  24 $$\begin{aligned} E_{Data(i)} &= K_{i} \times \{ [(E_{elec} + \varepsilon_{fs} r_{i}^{2} ) \times l_{1} + (n_{i} - 1) \times E_{elec} \times l + n_{i} \times E_{DA} \times l + (E_{elec} + \varepsilon_{fs} R_{t}^{2} ) \times l \hfill \\ &\quad + (2E_{elec} + \varepsilon_{fs} R_{t}^{2} ) \times l \times \frac{1}{{K_{i} }}\sum\limits_{j = i + 1}^{{L{}_{max}}} {K_{j} } ] + (n_{i} - 1) \times [E_{elec} \times l_{1} + (E_{elec} + \varepsilon_{fs} d_{toCH}^{2} ) \times l]\} \hfill \\ & = E_{elec} \times (l_{1} + l) \times N_{i} + E_{DA} \times l \times N_{i} + K_{i} \varepsilon_{fs} r_{i}^{2} \times l_{1} + K_{i} \varepsilon_{fs} R_{t}^{2} \times l \hfill \\ &\quad + (2E_{elec} + \varepsilon_{fs} R_{t}^{2} ) \times l \times \sum\limits_{j = i + 1}^{{L{}_{max}}} {K_{j} } + K_{i} \times (n_{i} - 1) \times (E_{elec} + \varepsilon_{fs} d_{toCH}^{2} ) \times l \hfill \\ \end{aligned}$$
  Data packets from ring *i* should be relayed to the sink with the help of inner rings, therefore, the additional energy consumption brought to the whole network by ring *i* can be obtained as follows:
  25 $$\Delta E_{Data} = (2E_{elec} + \varepsilon_{fs} R_{t}^{2} ) \times l \times K_{i} \times (i - 1)$$
- Analysis of the final results.
  The total energy consumption of ring *i E*_*total*(*i*)_ can be obtained by merging Eqs. (12), (15), (16) and (24), while the additional energy consumption brought to the whole network by ring *i* Δ*E*_*add*_ can be derived by merging Eqs. (21) and (25).
  26 $$\begin{aligned} E_{total(i)} &= 3(n_{i} \times E_{elec} + \varepsilon_{fs} r_{i}^{2} ) \times l_{1} \times N_{i} + K_{i} \times (n_{i} - 1) \times (2E_{elec} + \varepsilon_{fs} d_{toCH}^{2} ) \times l_{1} + K_{i} \times (E_{elec} \hfill \\&\quad + \varepsilon_{fs} R_{t}^{2} + E_{elec} \times K_{i - 1} \times P_{inRt} ) \times l_{1} + E_{elec} \times (l_{1} + l) \times N_{i} + E_{DA} \times l \times N_{i} + K_{i} \varepsilon_{fs} r_{i}^{2} \times l_{1} \hfill \\ &\quad + K_{i} \varepsilon_{fs} R_{t}^{2} \times l\; + (2E_{elec} + \varepsilon_{fs} R_{t}^{2} ) \times l \times \sum\limits_{j = i + 1}^{{L{}_{max}}} {K_{j} } + K_{i} \times (n_{i} - 1) \times (E_{elec} + \varepsilon_{fs} d_{toCH}^{2} ) \times l \hfill \\ \end{aligned}$$
  27 $$\Delta E_{add} = K_{i} \times E_{elec} \times l_{1} + (E_{elec} + \varepsilon_{fs} R_{t}^{2} ) \times l_{1} \times K_{i - 1} + (2E_{elec} + \varepsilon_{fs} R_{t}^{2} ) \times l \times K_{i} \times (i - 1)$$
  As we know, the number of CRSNs nodes in ring *i N*_*i*_ = *K*_*i*_ × *n*_*i*_ = *K*_*i*_ × π*r*_*i*_^2^*ρ*, and here *ρ* is the node density. According to^31^, the expected squared distance from a CM to its CH *ξ*[*d*_*toCH*_^2^] can be estimated by:
  28 $$\xi [d_{toCH}^{2} ] = \int_{\theta = 0}^{2\pi } {\int_{r = 0}^{{r_{i} }} {r^{2} \rho (r,\theta )drd\theta } } = \frac{{r_{i}^{2} }}{2}$$
  Our goal is to minimize the weighted sum of the energy consumption of ring *i* and the additional energy consumption brought to the whole network by it*.* The objective function can be expressed as:
  29 $${\text{Minimize}}\;(\alpha \times E_{total(i)} + \Delta E_{add} )$$
  where *α* is the weighted coefficient assigned to *E*_*total*(*i*)_. *E*_*total*(*i*)_ is closely related to the number of clusters *K*_*i*_. Under given topology, by minimizing the energy consumption of each ring *E*_*total*(*i*)_, we can achieve the goal of minimizing the network energy consumption and extending network lifespan. Δ*E*_*add*_ is the extra energy burden brought by ring *i* which is also related to the number of clusters *K*_*i*_, and if we can minimize the additional energy consumption brought to inner rings by each ring, the residual energy among nodes in different rings can be well balanced and the energy hole problem can be solved effectively. When the area of ring *i* and the total number of nodes in ring *i N*_*i*_ are given, as can be seen from Eqs. (26) and (27), increasing the number of clusters *K*_*i*_, *E*_*total*(*i*)_ will decline while Δ*E*_*add*_ will rise. In this case, the energy hole problem may occur and network surveillance capability will be negatively affected. On the contrary, decreasing *K*_*i*_, *E*_*total*(*i*)_ will increase while Δ*E*_*add*_ will reduce. The increase of *E*_*total*(*i*)_ will lead to faster node death in ring *i*, while the decrease of Δ*E*_*add*_ will lead to slower node death in inner rings. In this case, network lifetime will be shortened. In other words, there is a compromise between *E*_*total*(*i*)_ and Δ*E*_*add*_, i.e., there exists a reasonable *K*_*i*_ which can help avoid energy holes while extending network lifetime as much as possible. Therefore, by taking the derivative of Eq. (29) with respect to *K*_*i*_ and setting the result to 0, we can obtain the optimal number of clusters *K*_*i*_, and then we can obtain the optimal cluster radius *r*_*i*_ through Eq. (30).
  30 $$r_{i} = \sqrt {\frac{{N_{i} }}{{\pi \rho K_{i} }}}$$
  In addition, by combining Eqs. (11), (14), (16) and (22), the expected energy consumption of being a CH in ring *i E*_*CH*_*i*_ is shown in Eq. (31), and by substituting *R*_*t*_ with the actual Euclidean distance from node *j* to the sink, the obtained *E*_*CH_j*(*i*)_ can be used in Eq. (9).
  31 $$\begin{gathered} E_{CH\_(i)} = 3(n_{i} \times E_{elec} + \varepsilon_{fs} r_{i}^{2} ) \times l_{1} + E_{elec} \times l_{1} \times (n_{i} - 1) + (E_{elec} + \varepsilon_{fs} R_{t}^{2} ) \times l_{1} \hfill \\ \quad \quad \quad \quad \;\, + E_{elec} \times l_{1} \times K_{i - 1} \times P_{inRt} + (E_{elec} + \varepsilon_{fs} r_{i}^{2} ) \times l_{1} + E_{elec} \times l \times (n_{i} - 1) \hfill \\ \quad \quad \quad \quad \;\,+ E_{DA} \times l \times n_{i} + (E_{elec} + \varepsilon_{fs} R_{t}^{2} ) \times l + (2E_{elec} + \varepsilon_{fs} R_{t}^{2} ) \times l \times \frac{1}{{K_{i} }}\sum\limits_{j = i + 1}^{{L_{max} }} {K_{j} } \hfill \\ \end{gathered}$$

- The energy consumption of all nodes in ring *i* in CHs selection phase.
  In order to compete for CHs, each CRSNs node broadcasts its own information and ETBCHs value within cluster radius *r*_*i*_, and these information will be received by neighbors in the same ring. The corresponding energy consumption are (*E*_*elec*_ + *ε*_*fs*_*r*_*i*_^2^) × *l*_1_ and *E*_*elec*_ × *l*_1_ × (*n*_*i*_ − 1), respectively. *n*_*i*_ is the number of nodes within *r*_*i*_. By comparison, some powerful CRSNs nodes become CHs and broadcast CHs advertisement, while others become non-CHs nodes and broadcast quit messages. These messages will also be received by neighbors within *r*_*i*_. Therefore, no matter for a CH or a CM, the energy consumption in CHs selection phase can be represented by:
  11 $$e_{CHs} = 3(E_{elec} + \varepsilon_{fs} r_{i}^{2} ) \times l_{1} + 3E_{elec} \times l_{1} \times (n_{i} - 1) = 3(n_{i} \times E_{elec} + \varepsilon_{fs} r_{i}^{2} ) \times l_{1}$$
  Accordingly, the energy consumption of ring *i* in CHs selection phase is:
  12 $$E_{CHs(i)} = 3(n_{i} \times E_{elec} + \varepsilon_{fs} r_{i}^{2} ) \times l_{1} \times N_{i}$$
  where *N*_*i*_ is the number of CRSNs nodes in ring *i*.
- The energy consumption of ring *i* in cluster formation phase.
  Each non-CHs node, say node *j*, selects its own CH and sends out join request message. The CH receives the message and records *j* as its member. The energy consumption of a CM and a CH in cluster formation phase are shown in Eqs. (13) and (14), respectively.
  13 $$e_{Cluster\_CM} = (E_{elec} + \varepsilon_{fs} d_{toCH}^{2} ) \times l_{1}$$
  14 $$e_{Cluster\_CH} = E_{elec} \times l_{1} \times (n_{i} - 1)$$
  Consequently, the energy consumption of ring *i* in cluster formation phase is:
  15 $$\begin{gathered} E_{Cluster(i)} = (E_{elec} + \varepsilon_{fs} d_{toCH}^{2} ) \times l_{1} \times (n_{i} - 1) \times K_{i} + E_{elec} \times l_{1} \times (n_{i} - 1) \times K_{i} \hfill \\ \quad \quad \quad \quad \; = K_{i} \times (n_{i} - 1) \times (2E_{elec} + \varepsilon_{fs} d_{toCH}^{2} ) \times l_{1} \hfill \\ \end{gathered}$$
  where *K*_*i*_ is the number of CHs in ring *i*.
- The energy consumption of ring *i* and the additional energy consumption introduced to the whole network by ring *i* in route selection phase.
  Each CH in ring *i* − 1 broadcasts its own information within *R*_*t*_ to enable next-hop relay selection, and the information will be received by CHs in ring *i*. Each CH in ring *i* determines its next hop towards the sink and sends out routing notification. The corresponding inner CH receives this notification and becomes its next-hop relay. Consequently, the energy consumption of ring *i* in route selection phase is:
  16 $$E_{Route(i)} = K_{i} \times E_{elec} \times l_{1} \times K_{i - 1} \times P_{inRt} + (E_{elec} + \varepsilon_{fs} R_{t}^{2} ) \times l_{1} \times K_{i}$$
  where *K*_*i*−1_ is the number of CHs in ring *i* − 1, and *P*_*inRt*_ is the probability that an inner-ring CH is in the transmission range of ring-*i* CH. Therefore, *K*_*i*−1_ × *P*_*inRt*_ represents the excepted number of CHs in ring *i* − 1 from which ring-*i* CH can receive information. Fig. 5 is used to demonstrate how to calculate *P*_*inRt*_, and the detailed processes are as follows:

## Results and discussion

In this section, we test the performance of ECMUC protocol through extensive simulations by using Matlab which is one of the most commonly used simulation tools in evaluating the performance of clustering protocols designed for CRSNs^14,20,21,26^. In order to capture the impact of network size, we keep the node density *ρ* unchanged and analyze the performance of ECMUC protocol in three cases. In Case 1, *N* = 100 (*L*_*max*_ = 1) is picked to assess the performance of ECMUC when all CRSNs nodes can reach the sink through single-hop communication. In Case 2 and Case 3, *N* = 400 (*L*_*max*_ = 2) and *N* = 900 (*L*_*max*_ = 3) are picked to evaluate the performance of ECMUC with multi-hop inter-cluster relay. Other parameter settings are shown in Table 2.

**Table 2:** Simulation parameter settings.

Parameters	Values
Maximum node transmission range *R*_*t*_	50 m
Node density *ρ*	1/25π/m^2^
Initial energy of each CRSNs node *E*_0_	0.5 J
Energy consumption of transceiver electronics per bit *E*_*elec*_	50nJ/b
Energy consumption of amplifier in free-space loss model *ε*_*fs*_	10pJ/b/m^2^
Energy consumption of amplifier in multi-path loss model *ε*_*mp*_	0.0013pJ/b/m^4^
Energy consumption of data aggregation *E*_*DA*_	5nJ/b/packet
Data packet size *l*	1,000b
Control packet size *l*_1_	100b
Energy consumption per channel switching *E*_*sw*_	10 μJ
Number of PUs *P*	5
Total number of licensed channels *C*	5
Probability vector of ON states	[0.2, 0.3, 0.4, 0.5, 0.6]

ECMUC protocol is compared with current clustering protocols for CRSNs to verify its effectiveness, such as IMOCRP^14^, CogLEACH^15^, NSAC^16^, EACRP^17^, WCM^20^ and LEAUCH^21^. These protocols have been explained in the Related work section. Evaluation metrics, such as the number of living nodes at the end of round *t*, the ratio of effective data gathering nodes, the total control overhead and the number of selected CHs per round, are leveraged for performance comparison.

### Performance analysis in Case 1

**Figure 6:**
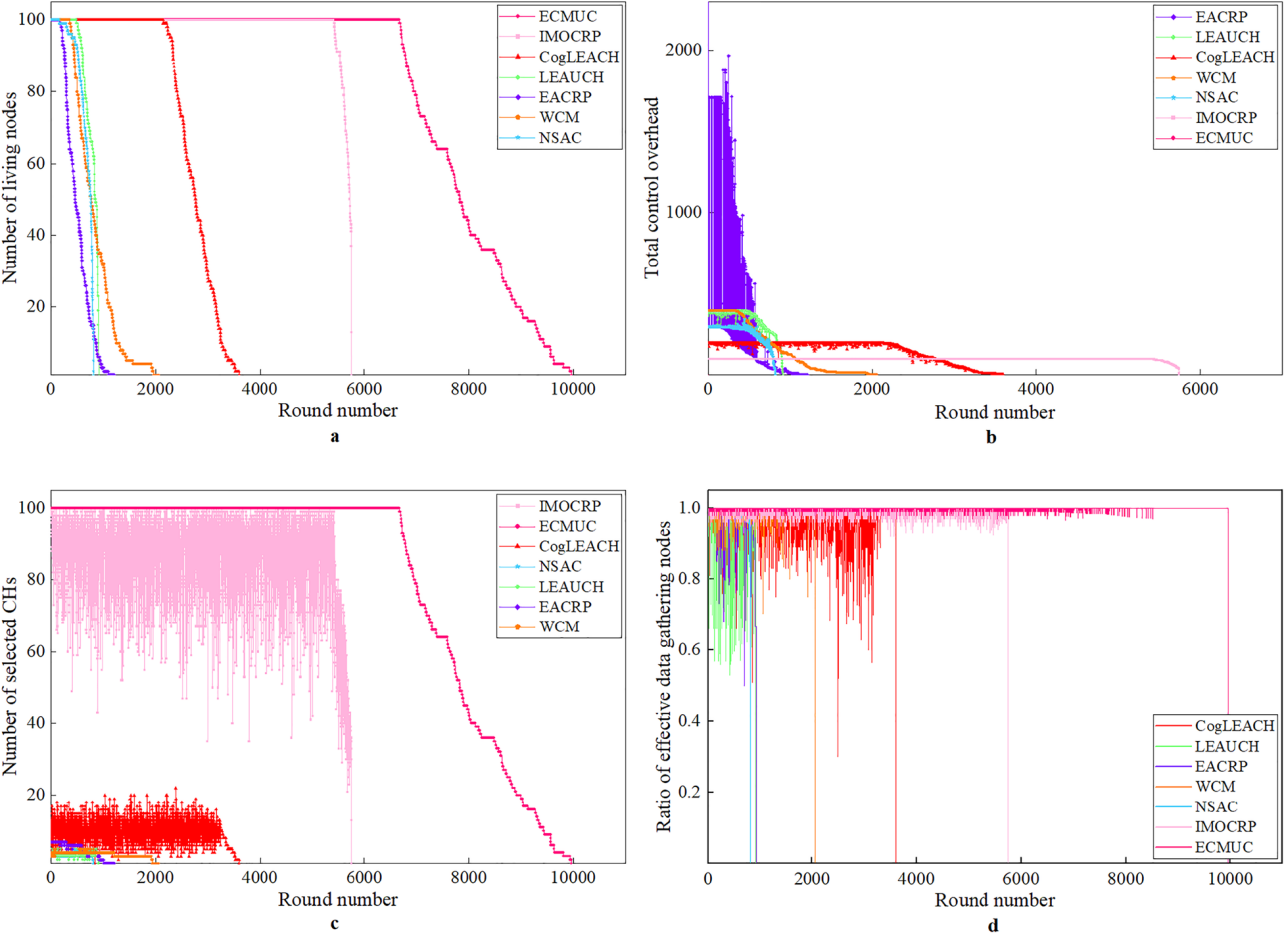
Performance comparison results in Case 1.

Performance comparison results with respect to above evaluation metrics in Case 1 are provided in Fig. 6. Among them, Fig. 6a shows the variations of number of living nodes with time (represented by round number) when these clustering protocols are applied. From it, we can observe that the number of living nodes of all competing protocols decreases as round number increases. CRSNs nodes are powered by limited-capacity battery, and tasks such as information transmission and reception, data aggregation and so on all consume energy and result in rapid reduction in residual energy. When the energy left in the battery is exhausted, the node is neglected from network operation and the number of living nodes decreases.

In order to compare these protocols explicitly, we record the rounds in which the first death node appears. Among these competitors, ECMUC protocol achieves the best performance. The first death node of ECMUC appears in round 6,668 which is much later than other protocols. The reasons can be analyzed from the sources of energy dissipation, i.e., the total control overhead and the number of selected CHs per round. Detailed analysis is listed below:

- As shown in Fig. 6b, the total control overhead of ECMUC in each round is always 0. In other words, there is no control information exchange among neighboring CRSNs nodes or between CRSNs nodes and the sink, which helps conserve energy. In this case, CRSNs nodes know that the sink is located within their communication range. They become separate CHs and directly deliver their monitored data to the sink. This explains why the number of selected CHs per round of ECMUC in Fig. 6c is obviously higher than its competitors, and this result is consistent with our theoretical analysis in Design details of ECMUC protocol section. Each living CRSNs node becomes an independent CH, which can result in the minimum network energy consumption. The only source of energy dissipation is data transmission from CRSNs nodes themselves to the sink, and their short Euclidean distance consumes a small amount of energy. Therefore, nodes can survive longer, which ensures strong network surveillance capability.
- As for IMOCRP, it is a centralized clustering protocol in which CHs selection is carried out by the sink instead of normal nodes. Therefore, each CRSNs node should transmit necessary information to the sink once per round, such as location or residual energy, that is, control overhead of CHs selection is incurred by information exchange between CRSNs nodes and the sink, not among neighboring CRSNs nodes. In IMOCRP, with the purpose of minimizing the weighted sum of the average node energy consumption and the standard deviation of node residual energy, the sink leverages intelligent algorithm to determine CHs and cluster membership. Therefore, its number of selected CHs is always higher than others except ECMUC. In addition, no information is exchanged among neighboring nodes for cluster formation and cluster channel selection, and the total control overhead of IMOCRP is equal to the number of living nodes at the beginning of that round. Thanks to its low control overhead, the first death node of IMOCRP appears in round 5,411. However, in Case 2 and Case 3, many CRSNs nodes cannot satisfy the single-hop communication requirement to the sink. Therefore, they cannot run IMOCRP effectively, and it is omitted from these two cases.
- Other protocols are distributed in nature, and their CHs selection and cluster formation rely on information exchange in the neighborhood. In CogLEACH, each CRSNs node can decide whether itself can become a CH or not by comparing its CHs weight with a randomly generated number (∈ [0,1]). Therefore, its control overhead for CHs selection is 0. However, it requires twice information exchange between CHs and normal nodes for temporary and final request and permission. The total control overhead of CogLEACH is about twice the number of living nodes at the beginning of each round. The random selection of CHs cannot guarantee the exact number and positions of optimal CHs, which can be observed from Fig. 6c. The number of CHs of CogLEACH in each round oscillates heavily. However, the optimal number of clusters used by CogLEACH is obtained by minimizing the total energy consumption of data transmission which is not quite comprehensive. This is the reason why their performance is worse than ECMUC. EACRP continuously merges neighboring clusters according to some predetermined rules until the termination condition is satisfied. A large number of control packets are exchanged during this process. Therefore, its total control overhead is very high. In addition, its control overhead is highly related to PUs activity, so dramatic oscillations can be observed from the total control overhead curve of EACRP in Fig. 6b. LEAUCH is an uneven clustering protocol. Its CHs selection is done through competition among candidate CHs, and its cluster formation process is the same as that of CogLEACH. Therefore, its total control overhead is usually smaller than four times the number of living nodes at the beginning of each round. It should be noted that candidate CHs in LEAUCH are selected by inheriting the CHs weight function from CogLEACH, and nodes whose weight is higher than the threshold can become candidate CHs. However, in Case 2 and Case 3, due to the high threshold defined by LEAUCH, none available candidate CHs can be picked out, not to mention the final CHs. Therefore, the performance of LEAUCH is omitted from Case 2 and Case 3. WCM and NSAC also require massive information exchange for CHs selection and cluster formation, so their number of living nodes reduces rapidly.

Apart from above performance metrics, we also care about the information collection capability each protocol can provide. This capability can be reflected by the ratio of effective data gathering nodes, and higher ratio means stronger capability. The comparison results among these protocols are shown in Fig. 6d. From it we can see that ECMUC can achieve the highest ratio of effective data gathering nodes. Each CRSNs node becomes a separate CH in ECMUC and it transmits data directly to the sink. The communication channel is randomly chosen from its available channel list, and the random channel selection decreases collision with PUs. Of course, channel reclaim from PUs is inevitable in ECMUC, as it only considers about the channel occupancy state at sensing time for simplicity purpose. If prediction of channel availability is included, the ratio of effective data gathering nodes can be further improved. As for other protocols, their curves of ratio of effective data gathering nodes oscillate heavily which depend on PUs activity. We can see from Fig. 6c that the number of clusters of these protocols is much fewer than that of ECMUC, and this means more CMs in each cluster. As common channels are required by each cluster, more CMs mean higher probability of collision with PUs, which will result in data transmission failures.

### Performance analysis in Case 2 and Case 3

**Figure 7:**
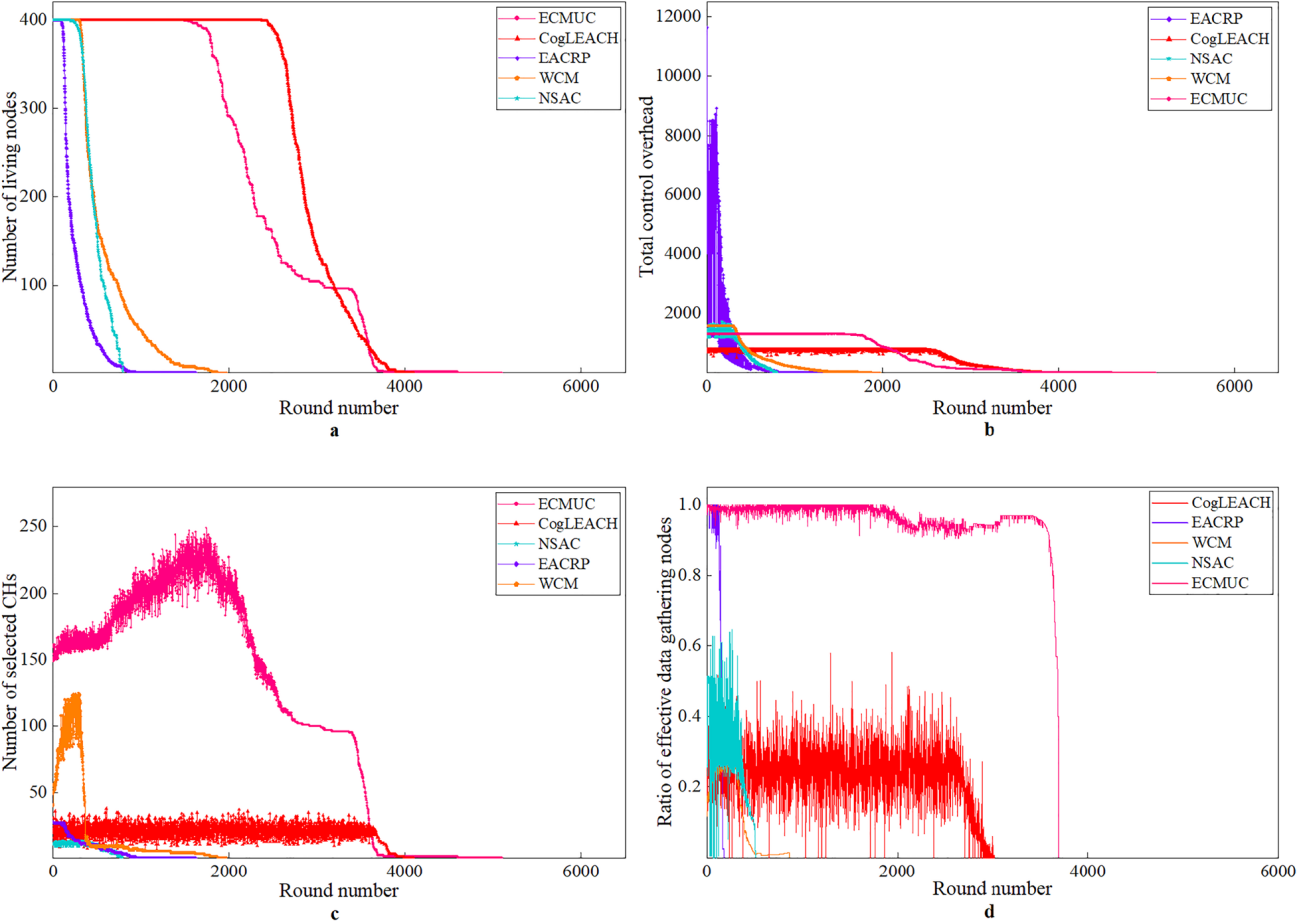
Performance comparison results in Case 2.

**Figure 8:**
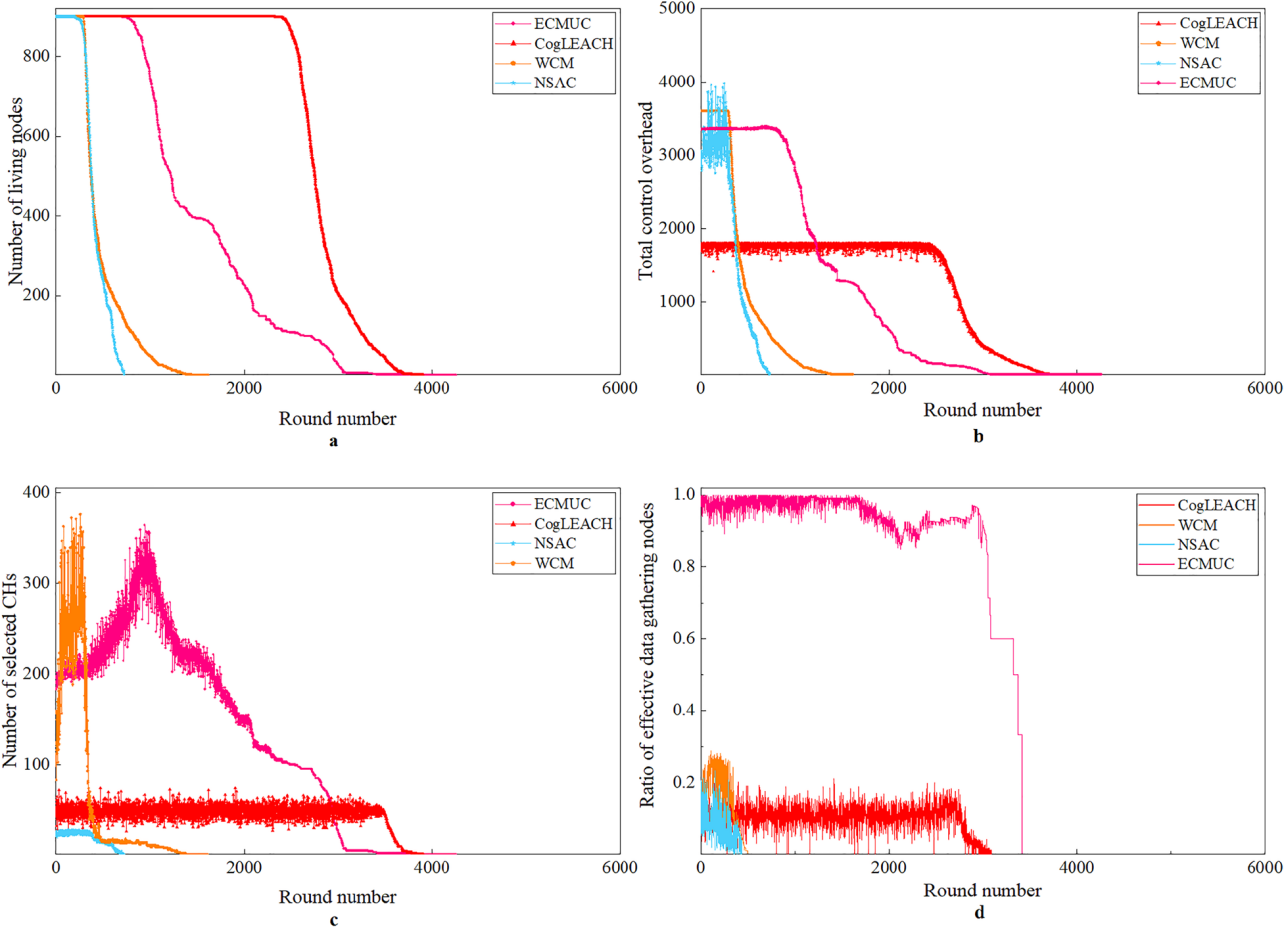
Performance comparison results in Case 3.

Case 2 and Case 3 are multi-hop communication scenarios which require inter-cluster relay. Performance comparison results in these two cases are shown in Figs. 7 and 8, respectively.

From Figs. 7 and 8, we can obtain the following observations:

- For ECMUC protocol, the time when the first death node appears is much later than EACRP, NSAC and WCM, but it is earlier than CogLEACH. The reasons are explained as follows: ECMUC is a multi-hop clustering routing protocol, and CRSNs nodes in it should consume extra energy to search for available routing paths and relay inter-cluster data packets. Therefore, compared with CogLEACH, the energy consumption of data packet transmission in ECMUC is higher, but thanks to its low energy consumption of control information exchange, its node death rate is slower after the first death node appears. From Figs. 7b and 8b, we can see that the total control overhead of ECMUC is heavier than CogLEACH, which means the number of control packets exchanged in ECMUC is more than that in CogLEACH. However, in ECMUC, the control information for CHs selection and cluster formation are both exchanged within the cluster radius which is much smaller than the maximum node transmission range *R*_*t*_, which helps reduce its energy consumed in control information exchange. Random CHs selection of CogLEACH results in the fluctuating number of CHs and their random positions, this may aggravate depletion of node energy. However, only CHs which can reach the sink through single-hop communication can successfully deliver their data, and this can help conserve energy to a certain extent. As for other protocols, especially EACRP, higher total control overhead of CHs selection and cluster formation together with routing overhead leads to faster node death. Its number of living nodes deteriorates rapidly.
- Similar to Case 1, the number of selected CHs in ECMUC is still much higher than its competitors, and it decreases as network operation goes on. In ECMUC, CRSNs nodes with the sink in their single-hop communication range automatically become CHs, which means each living CRSNs node in ring 1 is an independent CH. CHs competition in other rings is performed within cluster radius, which restricts the cluster size. Therefore, many small clusters are formed in ECMUC, which is beneficial for conserving energy. In EACRP, neighboring clusters can be merged only if any two nodes in these clusters are within each other’s transmission range. This illustrates why more clusters are formed in EACRP than in CogLEACH and NSAC under the same network conditions.
- As shown in Figs. 7d and 8d, the ratio of effective data gathering nodes of ECMUC is much higher than other competing protocols. As a multi-hop clustering routing protocol, ECMUC relies on effective intra-cluster data collection and inter-cluster relay to forward data packets towards the sink. It determines reasonable routing paths for packet delivery. Therefore, most data packets can reach the sink successfully. Similarly, living CRSNs nodes in EACRP can transmit their data successfully to the sink through the multi-hop forwarding paths which are found during route selection process, and this explains why EACRP can achieve high ratio of effective data gathering nodes at the beginning of network operation. However, fast energy exhaustion leads to poor network surveillance capability which cannot satisfy requirements of practical applications. CogLEACH, WCM and NSAC can only achieve low ratio of effective data gathering nodes. The reason is that they are all designed for scenarios which require single-hop communication between CHs and the sink, so they consider nothing about inter-cluster routing. Due to the limited node transmission range and long distance between CRSNs nodes and the sink in Case 2 and Case 3, data packets from many nodes cannot reach the sink at all.
- By comparing Figs. 6 with 7 and 8, we can observe the impact of number of nodes on network performance. In general, when the node density *ρ* is kept unchanged, more nodes will lead to shorter network lifespan, higher total control overhead, higher number of selected CHs in each round and a little lower ratio of effective data gathering nodes. In order to compete for CHs in the neighborhood and form clusters, each CRSNs node *j* (*L*(*j*) ≠ 1) needs to broadcast or unicast related information, that is, the total control overhead is positively proportional to the number of living nodes. In addition, more nodes in outer rings will increase the relay burden of nodes in inner rings. Even though the energy consumption of control information exchange can be effectively controlled by reasonably setting the cluster radius, heavier inter-cluster data relay will accelerate the energy exhaustion of nodes in inner rings. In this case, network lifetime will be shortened.

As demonstrated above, in Case 1, ECMUC can achieve the best performance among all competing clustering protocols. All living CRSNs nodes automatically become independent CHs and transmit data directly to the sink. No control information is exchanged among CRSNs nodes, which results in the minimum network energy consumption. In Case 2 and Case 3, ECMUC is still superior to other competing protocols in terms of the ratio of effective data gathering nodes, which means ECMUC can guarantee strong network surveillance capability. However, its number of living nodes metric is inferior to CogLEACH, and this is the cost paid for route selection and multi-hop inter-cluster data relay. However, there are much more effective data gathering nodes in ECMUC during long-term network operation. In other words, ECMUC can provide powerful surveillance capability while compromising the lifespan of a portion of nodes.

## Conclusions

Previous clustering protocols for CRSNs only consider about minimizing the total energy consumption of data communication and omit the impact of control overhead. Aiming at conquering this limitation and solving the multi-hop inter-cluster routing problem in large-scale CRSNs, an uneven clustering routing protocol ECMUC is proposed in this paper. ECMUC combines energy consumption of data communication and control information exchange together for the first time to form the first part of the clustering objective. Then minimizing the network energy consumption and balancing the residual energy among nodes in different rings are innovatively transformed into minimizing the weighted sum of the energy consumption of each ring and the additional energy consumption introduced to the whole network by it. Cluster radiuses of different rings are theoretically derived, and they control the cluster size as well as the corresponding energy consumption of control information exchange. ECMUC applies varied CHs selection and cluster formation strategies to different rings, and it selects nodes with the greatest energy and spectral potential as CHs. Through massive well-designed simulations, we demonstrate that control overhead has a large impact on energy consumption, and taking it into consideration is beneficial for improving network performance. In addition, ECMUC is superior to most current clustering protocols in terms of network surveillance capability and network lifetime. In the future, we will leverage energy harvesting and simultaneous wireless information and power transfer technology to further prolong network lifespan. In addition, we will research on how to determine the cluster radius automatically according to specific network configurations.

## Data Availability

The datasets generated during and/or analysed during the current study are available from the corresponding author on reasonable request.
